# The impact of rural collective property rights reform on income and poverty reduction: Evidence from China’s rural regions

**DOI:** 10.1371/journal.pone.0308393

**Published:** 2024-09-06

**Authors:** Qin Xiang, Jingjin Li, Guoyong Liu

**Affiliations:** College of Economics and Management, Xinjiang Agricultural University, Urumqi, China; Shandong University of Technology, CHINA

## Abstract

The reform of rural collective property rights is pivotal in advancing agricultural modernization and comprehensive rural revitalization. This study aims to explore the impact of this reform on income growth and poverty reduction in rural areas, as well as its underlying mechanisms. Utilizing data from the China Rural Revitalization Survey (CRRS), the propensity score matching (PSM) method was employed to empirically analyze the effects of rural collective property rights reform on income growth and poverty reduction. The findings reveal that the reform has a significant positive impact on rural income levels, indirectly enhancing income through two channels: increasing village collective assets and promoting land transfer. Additionally, the reform has a significant negative impact on rural poverty levels, effectively alleviating poverty in rural areas. Heterogeneity analysis further reveals the differential effects of property rights reform, indicating that non-poor villages, villages with higher educational levels among village leaders, and rural areas in the western regions benefit more from the reform. This study provides precise evidence for policymakers, offering a scientific basis for deepening rural collective property rights reform, promoting income growth, and fostering sustainable rural development.

## Introduction

The reform of the rural collective property rights system signifies a profound transformation in the development of China’s rural areas, playing a crucial role in optimizing resource allocation, invigorating rural vitality, and ensuring farmers’ rights [1]. The government places great importance on this reform, having introduced a series of policies to clarify property ownership, activate rural assets, and protect farmers’ rights [2]. The issuance of the ’Opinions on Comprehensively Deepening Rural Reform and Accelerating the Advancement of Rural Modernization’ in 2014 underscored the importance of the rural collective property rights system reform [3]. In 2020, the Ministry of Agriculture and Rural Affairs released the ‘Guiding Opinions on Deepening the Reform of the Rural Collective Property Rights System,’ which explicitly called for the acceleration of the rural collective assets’ inventory verification to clarify ownership, enhance the construction of rural collective economic organizations, and foster the standardized management and transparent operation of collective assets [4]. The State Council’s ’Opinions on Deepening Rural Reforms to Stimulate the Vitality of Rural Development’ released in 2021 emphasized the need to improve the rural collective property rights system, advance the reform of collective operational assets into a shareholding cooperation system, develop diversified forms of rural collective economies, and strengthen the economic power of rural collectives [5]. The reform of rural collective property rights in China has successfully invigorated the rural economy by clarifying land and asset ownership. For example, since the implementation of the reform in Zhejiang Province, the land transfer rate has increased by 30%, and the annual income of village collective economies has grown by nearly 50% [6]. Farmers, through the establishment of land contracting rights, have not only enhanced their investment and management of land but also gained additional property income through the transfer market. In Jiangsu Province, over 60% of farmers have benefited from dividends on collective assets by participating in cooperatives, with an average annual income increase of about 20% [7]. Additionally, the reform has promoted the optimization and upgrading of the agricultural industrial structure. For instance, in Sichuan Province, agricultural output has achieved an average annual growth of 15% through the introduction of modern agricultural technologies and management [8].

The Rural Collective Property Rights Reform by clarifying the ownership of land and collective assets, has laid a foundation for the healthy development of the rural collective economy, marking a crucial step towards agricultural modernization and the increase of farmers’ income [9]. Throughout the development journey of China’s rural collective economy, the ambiguity of property rights has always been a primary hindrance to progress [10]. The lack of stable legal protection for farmers’ rights to use land and collective assets directly impacts their willingness to invest in agricultural production and rural economic activities [11]. With the reform of property rights, the land contracting rights and operational rights of farmers have been strengthened, and the land transfer market has been standardized [12], thus facilitating the efficient utilization of land resources and the diversified development of the rural economy. This not only enhances the efficiency of agricultural production but also provides farmers with more opportunities for non-agricultural employment and entrepreneurship [13]. Moreover, the Rural Collective Property Rights Reform, by activating “dormant” rural assets and improving the efficiency of asset operation, has increased farmers’ property income and dividend earnings [14]. As the reform progresses, the developmental potential of the rural collective economy has been unleashed, elevating the economic and social status of farmers, optimizing the rural economic structure, and providing a solid economic foundation for the implementation of the Rural Revitalization Strategy [15]. In summary, Rural Collective Property Rights Reform is a vital pathway to rural economic development, societal progress, and the enhancement of farmers’ welfare [16], exerting a profound influence on steering China’s countryside towards comprehensive rejuvenation and sustainable development. As a significant component of China’s rural reforms, it remains a focal point of academic interest. Research by international scholars on rural collective property rights reform has primarily focused on the confirmation of land rights, the reform of land contract management rights, the efficiency of collective action and resource management, and the flexibility and dynamism of property rights systems. Lawry (2017) systematically reviewed the impact of land tenure reforms on investment and agricultural productivity in developing countries. It was found that land tenure reforms generally lead to increased agricultural investment and enhanced productivity. However, the effects vary depending on specific social, economic, and institutional contexts [17]. Hartvigsen (2014) examined land reform and land fragmentation issues in Central and Eastern Europe, proposing two basic methods for the restoration and allocation of land rights [18]. Rodgers (2009) discussed property rights, land use, and reform issues in rural environments, suggesting the need for a flexible and dynamic common property rights system to address challenges in rural settings [19]. Lipton (2009) analyzed land reform in developing countries, highlighting that the inequality of land rights and the organizational capacity of affected groups are critical factors for the success of land reforms. Bu and Liao (2022) investigated the impact of land property rights on the growth of rural enterprises, finding that land titling reform helps promote the development of rural enterprises [20]. Totin et al. (2021) studied land reform in Mali, emphasizing the role of collective property in sustainable food production [21]. Coulibaly (2021) argued that land transactions help ensure individual or collective land rights [22]. Scholars have extensively discussed the impacts of reforms from various perspectives, including the optimization of property structures, the improvement of land transfer markets, the enhancement of agricultural efficiency, and the improvement of rural governance structures. However, despite the extensive coverage of various aspects of rural collective property rights reform, research on its income-increasing and poverty-reducing effects remains relatively scarce, particularly in terms of micro-level empirical analysis.

This study, by conducting an in-depth analysis of the data from the China Rural Revitalization Survey (CRRS), uncovers the significant impact of the reform of the rural collective property rights system on the growth of farmers’ income and the reduction of poverty. The marginal contributions of this paper are as follows: First, this paper utilizes the latest micro-level data, which encompasses a wider geographical range and a more diverse socio-economic background, thereby offering a more holistic perspective. Second, the research findings reveal that the reform of the property rights system has a significant positive effect on increasing farmers’ income levels through two key channels: increasing the total assets of village collectives and promoting land transfer. Third, the discoveries of this paper not only provide new evidence for understanding the complex mechanisms of property rights reform but also offer a scientific basis for the formulation of more effective rural development policies.

## Theoretical analysis and research hypothesis

**Fig 1:**
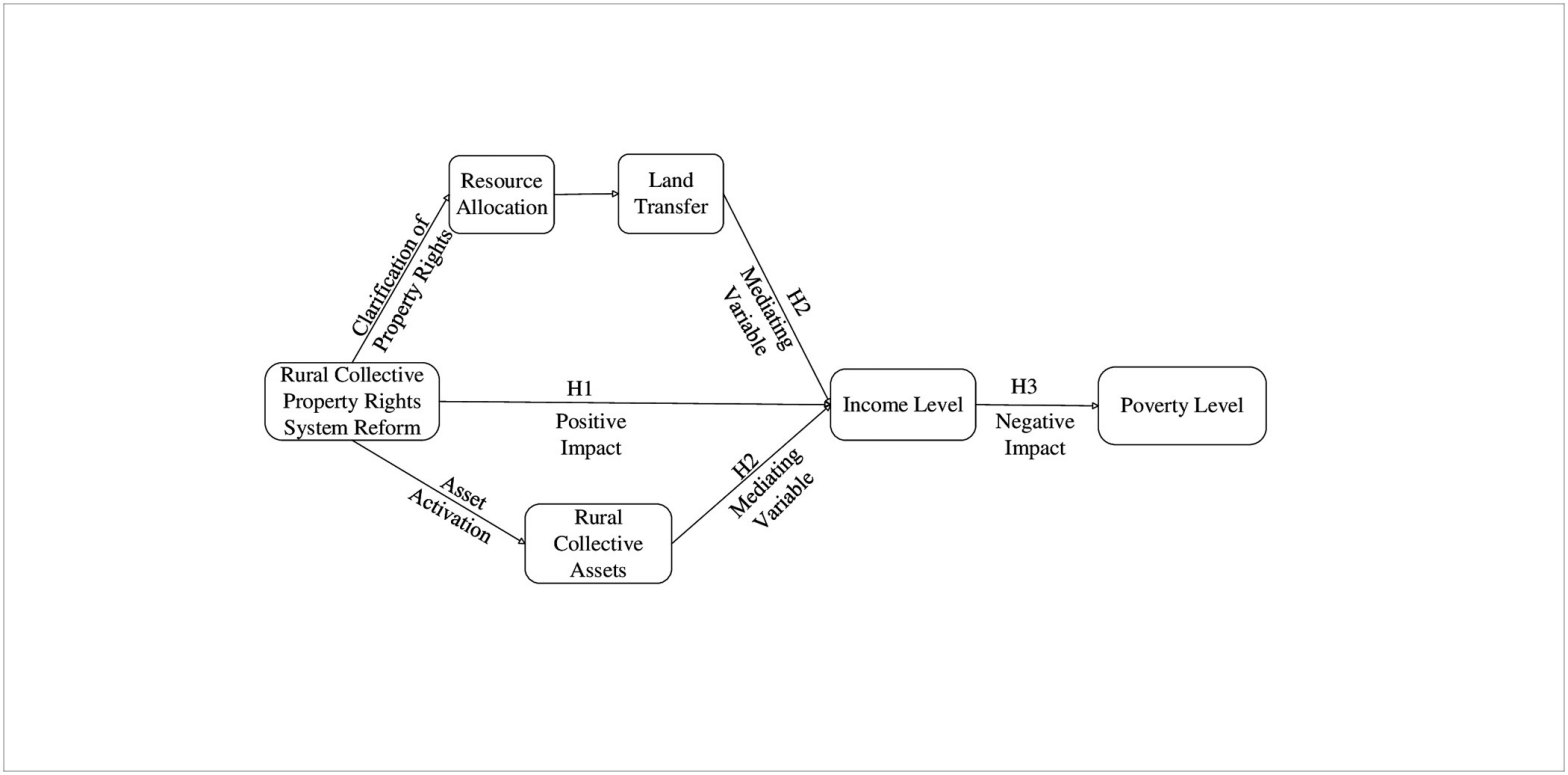
The theoretical framework for the income-increasing and poverty-reducing effects of rural collective property rights system reform.

Against the backdrop of the rural collective property rights system reform in China, it is particularly crucial to explore its theoretical analysis and the effects on income increase and poverty reduction. The reform of the rural collective property rights system, as a deepened innovation of the rural economic system [23], enhances farmers’ organized involvement and market participation at the societal level. Consequently, at the micro level, it stimulates agricultural households’ enthusiasm for production and innovation capabilities. At the macro level, it promotes the rational allocation of resources and the efficient integration of the agricultural industry chain. This results not only in improved agricultural production efficiency and increased income but also drives the comprehensive development of the rural economy and the sustained income growth of farmers. Fig 1 illustrates the theoretical framework for the income-increasing and poverty-reducing effects of rural collective property rights system reform.

The reform of the rural collective property rights system is a key initiative during China’s economic transformation period, aimed at promoting the development of productive forces and enhancing income levels by adjusting and optimizing rural production relations [24]. Property rights theory underscores that the clarification and tradability of property rights are critical factors in stimulating farmers’ enthusiasm for production [25]. Clear and stable property relationships can reduce transaction costs and minimize the excessive consumption and waste of resources, thereby enhancing the efficiency of resource allocation [26]. This theory provides theoretical underpinning for the first hypothesis of this paper, namely, that farmers, with stable use-rights and income rights to land through reforms in rural collective property rights, are inclined to carry out long-term improvements and technological innovations on the land. These reforms and innovative activities not only enhance the output efficiency of the land but also promote the development of the rural economy and the growth of farmers’ incomes. Consequently, this paper proposes the following hypothesis:

H1: Rural collective property rights system reform has a positive effect on income levels.

The reform of the rural collective property rights system, by affirming farmers’ land use and management rights, has invigorated the land transfer market, enabling more efficient allocation of land resources. This has enhanced the scale and efficiency of agricultural production, thereby directly increasing agricultural income [27]. Moreover, the reform has bolstered the asset management capability of rural collective economic organizations, augmenting the total value of collective assets. Through the provision of public services and improvements in infrastructure, non-agricultural income has been indirectly increased [28]. In this process, the transfer of land and the augmentation of rural collective economic assets serve as mediating variables, reinforcing the positive correlation between property rights system reform and income levels. This unveils the multifaceted mechanisms through which reforms in rural collective property rights bolster the transformation of the rural economic structure and income growth. Hence, the following hypothesis is proposed:

H2: Land transfer and rural collective economic assets play a mediating role between property rights system reform and income increase.

The theoretical construct regarding the poverty alleviation effect of rural collective property rights system reform synthesizes poverty trap theory, social capital theory, and developmental economics perspectives. This framework posits that reform, by endowing farmers with land use and operational rights [29], provides an asset foundation to break the cycle of poverty, enabling farmers to increase income through agricultural production and other economic activities. Furthermore, the reform fortifies rural collective economic organizations and cooperative mechanisms, fostering mutual assistance, cooperation, and information exchange among farmers, accruing social capital, increasing collective action capabilities, collectively addressing challenges in production and daily life, and mitigating poverty caused by information asymmetry and market access barriers [30]. Although an increase in income levels does not always directly lead to a reduction in poverty levels, income inequality can result in the impoverished population not benefiting from overall income growth [31, 32]. Zhou (2023) have demonstrated that reforms can directly and indirectly affect poverty levels by enhancing social capital and improving governance structures [33]. Additionally, the reforms promote the optimization and upgrading of the agricultural industrial structure, increase the added value of agriculture, provide more employment opportunities for farmers, and encourage the development of non-agricultural industries. This, in turn, promotes diversified employment and contributes to the reduction of rural poverty from the perspective of economic structural changes [34]. Thus, the following hypothesis is posited:

H3: The reform of rural collective property rights has a significant negative impact on poverty levels by improving infrastructure and public services, and enhancing social capital.

## Research design

### Model specification

Baseline Regression Model [35]: Pursuant to the theoretical analysis outlined above, the following regression model is constructed:

$$Reg\left(I\text{ncome}\right)=\beta_{0}+\beta_{1}Reform_{ij}+\beta_{2}X_{ij}+\varepsilon_{ij}$$ (1)

$$Reg\left(P\text{oor}\right)=\beta_{0}+\beta_{1}Reform_{ij}+\beta_{2}X_{ij}+\varepsilon_{ij}$$ (2)

The expressions *Reg* (*I*ncome) and *Reg* (*P*oor) represent regression models for rural income levels and poverty rates, respectively. "*Income*" denotes rural income levels, while "*Poor*" signifies the poverty rate. *β*_0_ is the intercept term, representing the expected value of the dependent variable (rural income level or poverty rate) when all independent variables (*Reform*_*ij*_ and *X*_*ij*_) are zero. *β*_1_ is the coefficient term. *Reform*_*ij*_ represents the rural economic reform variable for the *i*-th observation in the *j*-th village. *β*_2_ is the coefficient term that reflects the impact of the control variables *X*_*ij*_ on the dependent variable. *X*_*ij*_ are the control variables, encompassing other factors that might influence rural income levels or poverty rates, apart from the reform. *ε*_*ij*_ is the error term, capturing the random variation or measurement error that the model cannot explain.

Mediation effect model [36, 37]:

$${\text{Income}}_{ij}{=\text{c}}_{0}{+\text{c}}_{ij}{\text{Reform}}_{i}+{\text{c}}_{2}X_{ij}+\varepsilon_{ij}$$ (3)

$$M_{ij}=\alpha_{0}+\alpha_{1}Reform_{ij}+\alpha_{2}X_{ij}+\phi_{ij}$$ (4)

$$Income_{ij}=b_{0}+\text{c}\prime Reform_{ij}+b_{1}M_{ij}+b_{2}X_{ij}+\omega_{ij}$$ (5)

Within the framework, Income_*ij*_ is designated as the dependent variable, representing the level of rural income. Reform_*ij*_ pertains to the reform of rural collective property rights, with *M*_*ij*_ signifying the mediating variables. *X*_*ij*_ encompasses a suite of control variables. Moreover *α*_0_, *b*_0_ and *c*_0_ signify constant terms. The coefficients awaiting estimation are represented by *α*_1_, *b*_1_, c′, *c*_1_, *α*_2_, *b*_2_ and *c*_2_, whilst *ε*_*ij*_, *ϕ*_*ij*_ and *ω*_*ij*_ constitute the error terms.

### Variable selection

#### Dependent variables

The dependent variables primarily appertain to the economic conditions in rural areas and encompass two aspects: firstly, the level of rural income, which is expressed as the logarithm of per capita disposable income to measure the overall economic level in rural areas; secondly, the rural poverty rate, which is reflected by the proportion of the impoverished population relative to the total population.

#### Independent variable

The completion status of rural collective property rights reform serves as the independent variable, aimed at investigating the impact of whether the reform has been carried through on the economic conditions in rural areas. This variable is binary in nature, with the value of 1 denoting that the reform has been completed, while a value of 0 indicates the reform is pending completion.

#### Mediating variables

Mediating variables function as conduits in the model, linking the independent and dependent variables. This study includes two mediating variables: land circulation and village collective assets. Land circulation is gauged by the area of the transferred land (in mu), and village collective assets are measured by the amount of the total collective assets (in ten thousand yuan). Both are poised to potentially mediate the impact of property rights reform on the rural economy.

#### Control variables

Control variables are employed to account for other factors in the model that may influence the results. The control variables in this study consist of the average age of village party organization members, the total number of party members in the village, the workforce in the primary sector, the rural labor force, operational expenditures, public welfare expenditures, the distance from the village committee to the county government, the proportion of road construction and maintenance in rural areas, and the geographical location of the rural area. These variables, covering social structure, economic activity, infrastructure, and geographical position, contribute to a more accurate assessment of the impacts of rural collective property rights reform on the rural economy. Table 1 presents the descriptive statistics for all variables.

**Table 1:** Variable definitions, measurements, and descriptive statistical analysis.

Variable Name	Variable Symbol	Mean	Standard Deviation	Minimum	Maximum
Rural Income Level	Income	9.427	0.732	0.693	11.775
Rural Poverty Rate	Poor	0.179	0.154	0.004	0.939
Rural Collective Property Rights Reform	Reform	0.624	0.485	0	1
Land Transfer	M_1_	1024.736	1453.29	0	8000
Village Collective Assets	M_2_	573.209	734.160	0	3662
Average Age of Village Party Organization	X_1_	51.237	6.194	30	69.3
Total Number of Party Members in the Village	X_2_	52.601	32.474	5	194
Number of People in the Primary Industry	X_3_	515.879	509.839	0	2896
Number of Rural Labor Force	X_4_	1314.182	1074.954	70	7600
Operational Expenditure	X_5_	11.949	43.131	0	560
Public Welfare Expenditure	X_6_	3.782	13.963	0	169
Distance from the Village Committee to the County Government	X_7_	2.852	0.817	0	4.83
Proportion of Rural Road Construction and Maintenance Expenditure	X_8_	0.141	0.218	0	1
Rural Location	X_9_	0.217	0.413	0	1

### Data source

The data for this study were derived from a comprehensive national rural longitudinal survey initiated and carried out by the Rural Development Institute of the Chinese Academy of Social Sciences, dubbed the "China Rural Revitalization Survey" (hereinafter referred to as CRRS). Anchored in the significant socio-economic survey project of the Chinese Academy of Social Sciences, "Comprehensive Survey for Rural Revitalization and Construction of Chinese Rural Survey Database," the CRRS encompasses pivotal aspects of rural development such as "rural population and labor force," "rural industrial structure," "farmers’ income and expenditure & social welfare," "rural residents’ consumption," "rural governance," and "comprehensive rural reform." The survey was conducted across ten provinces and autonomous regions—including Guangdong, Zhejiang, Shandong, Anhui, Henan, Heilongjiang, Guizhou, Sichuan, Shaanxi, and Ningxia Hui Autonomous Region—encompassing 50 counties (cities) and 156 townships (towns), accruing 300 rural survey questionnaires and more than 3,800 household survey questionnaires, amassing information on over 15,000 family members. The data employed in this paper are from the initial phase of the CRRS, which commenced in the year 2020, with a primary focus on rural survey data.

## Empirical analysis

### Baseline regression results

The baseline regression outcomes, as presented in Table 2, reveal that columns (1) and (2) exhibit the effects of rural collective property rights reform on income levels after progressively including core independent variables and control variables. The results indicate that the reform of rural collective property rights has a significant positive influence on rural income levels, thereby validating Hypothesis 1. The reform’s resource optimization, enhanced incentive mechanisms, and clarified property rights collectively contribute to the economic development and income growth in rural areas. Among the control variables, the average age of the village party organization, total number of village party members, public welfare expenditures, and rural locality have a notable positive effect on rural income levels. This suggests that villages located in suburban areas, those with an older average age of village party organization members, a larger number of party members, and greater public welfare spending typically have higher income levels. The underlying reasons could be attributed to the suburban areas’ ease of access to the economic benefits emanating from urban centers, the belief that the older average age of village party organizations may correlate with more experience and stability, an increase in party members reflecting stronger organizational capacity and policy enforcement, and higher public welfare spending correlating with improvements in rural public services and infrastructure development. All of which are conducive to elevating the general economic standard of rural areas. The number of individuals working in the primary sector has a significant negative impact on rural income levels, suggesting that a larger labor force in the primary sector could be inversely related to rural income. This could be due to the typically lower production efficiency of the primary sector and its vulnerability to natural conditions like weather, which limits income growth potential. Columns (3) and (4) disclose the effects on rural poverty levels after a step-by-step introduction of core independent variables and control variables. The findings demonstrate that rural collective property rights reform significantly reduces the level of rural poverty, implying that such reforms can effectively mitigate the severity of rural poverty. Possible mechanisms include increased efficiency in resource utilization, a rise in property-based income for farmers, and promotion of non-agricultural employment. Among control variables, the distance between the villa committee and the county government significantly raises rural poverty levels. Villages further from the county center likely struggle to obtain governmental resource allocation and policy support, with poor accessibility potentially hampering economic initiatives and external investments. Meanwhile, the rural location shows a significant negative effect on the poverty level, indicating that rural areas in suburban regions tend to have lower poverty levels compared to those outside suburban areas, possibly due to their proximity to urban economies and access to better infrastructure and public services, which provides more employment opportunities and economic growth.

**Table 2:** Baseline regression results.

	Income		Poor	
Variable Name	(1)	(2)	(3)	(4)
Reform	0.211***	0.138***	-0.071***	-0.069***
	(0.056)	(0.053)	(0.019)	(0.019)
X1		0.0001***		-0.0002
		(0.0001)		(0.001)
X2		0.003*		-0.0007
		(0.001)		(0.0005)
X3		-0.0001**		-0.0001
		(0.0001)		(0.0002)
X4		-0.00003		0.0001
		(0.0001)		(0.0001)
X5		-0.0004		0.0001
		(0.0006)		(0.0002)
X6		0.004**		-0.0001
		(0.002)		(0.0007)
X7		-0.057		0.054***
		(0.037)		(0.013)
X8		0.012		-0.042
		(0.116)		(0.042)
X9		0.129*		-0.050**
		(0.067)		(0.024)
R^2^	0.0518	0.2653	0.0502	0.1895

Note:

*, **, and *** respectively indicate significance at the 10%, 5%, and 1% statistical levels. The standard errors are indicated within the parentheses. This notion will be followed subsequently.

Thus, the reform of rural collective property rights plays a positive role in improving the economic level and reducing the poverty rate in rural areas. Furthermore, factors such as the geographical position of rural areas, organizational structure, and public expenditure also significantly influence the rural economic situation. These findings provide sound evidence for the formulation of targeted rural development policies.

### Robustness check

Trimming of Extremes: To address potential extremities and outliers within the sample data, the study employed a tail-trimming method at the 5% percentile. The regression outcomes post this treatment are displayed in Table 3, which illustrates that the reform of rural collective property rights has a significant positive effect on rural income levels (as shown in columns (1) and (2)). With every additional unit in the depth of reform, the average rural income level increased by 0.318 log units (Model 1) and 0.271 log units (Model 2). Concurrently, the reform of rural collective property rights also exhibits a significant negative impact on levels of rural poverty (as indicated in columns (3) and (4)), where an increase in the depth of reform correlates with an average reduction of 0.071 percentage points in the poverty rate (Models 3 and 4). These findings signify that the positive effects of the reform of rural collective property rights on the rural economy remain robust even after the exclusion of extreme values and anomalies.

**Table 3:** Censored data processing results.

Variable Name	Income		Poor	
	(1)	(2)	(3)	(4)
Reform	0.318***	0.271***	-0.071***	-0.071***
	(0.092)	(0.091)	(0.019)	(0.019)
X1		0.019***		0.0002
		(0.007)		(0.001)
X2		0.002		-0.0007
		(0.002)		(0.0004)
X3		-0.0001**		-0.00001
		(0.0001)		(0.0001)
X4		-0.0001		0.0001
		(0.0006)		(0.0001)
X5		-0.0004		0.0001
		(0.001)		(0.0002)
X6		0.003**		-0.0001
		(0.003)		(0.0007)
X7		-0.108*		0.047***
		(0.059)		(0.012)
X8		-0.009		-0.042
		(0.203)		(0.042)
X9		0.215*		-0.048**
		(0.118)		(0.024)
R^2^	0.0445	0.1469	0.0502	0.1847

Alternative Independent Variables: In a bid to further verify the robustness of the findings, researchers employed the depth of rural collective property rights reform as a metric for gauging the impact of reform. This included considerations on whether property rights reform was undertaken, whether collective share equity was established, and whether dividends from collective shareholdings were distributed. An affirmative answer to each query contributed one point towards the reform depth score. The results, as delineated in Table 4, reveal that the depth of rural collective property rights reform has a significant positive effect on rural income levels (columns (1) and (2)). For every unit increase in reform depth, there is an average increase of 0.089 log units (Model 1) and 0.071 log units (Model 2) in rural income levels. Regarding rural poverty levels, an elevation in reform depth also led to a notable decrease in the poverty rate (columns (3) and (4)), with an average reduction of 0.071 percentage points (Model 3) and 0.031 percentage points (Model 4) in poverty rates. Therefore, Hypothesis 2 is substantiated.

**Table 4:** Results of independent variable substitution.

Variable Name	Income		Poor	
	(1)	(2)	(3)	(4)
Reform	0.089***	0.071**	-0.071***	-0.031***
	(0.029)	(0.028)	(0.019)	(0.010)
X1		0.012***		0.0001
		(0.004)		(0.001)
X2		0.003**		-0.0007
		(0.001)		(0.0004)
X3		-0.0001**		-0.0001
		(0.0001)		(0.0001)
X4		-0.0001		0.0001
		(0.0004)		(0.0001)
X5		-0.0004		0.0001
		(0.001)		(0.0002)
X6		0.004**		-0.0001
		(0.002)		(0.0007)
X7		-0.058		0.054***
		(0.037)		(0.013)
X8		-0.003		-0.037
		(0.120)		(0.042)
X9		0.163**		-0.049**
		(0.070)		(0.024)
R^2^	0.0358	0.2019	0.2019	0.1786

In terms of control variables, both the average age of village party organizations and the total number of village party members exhibited a significant positive impact in the regression analyses concerning rural income levels. Conversely, the number of people working in the primary sector demonstrated a significant negative effect on rural income levels. Operational expenditures and public welfare expenditures had negligible impacts on rural poverty levels. Meanwhile, the distance from the village committee to the county government showed a significant positive effect on rural poverty levels, indicating that villages further from the county center experienced higher levels of poverty. The location of rural areas had a significant negative impact on poverty levels, suggesting that poverty levels in suburban rural areas were comparatively lower. Thus, whether through tail trimming or the substitution of independent variables, the robustness checks support the conclusion that the reform of rural collective property rights positively affects raising rural income levels and reducing poverty levels. These checks bolster the credibility of the study’s outcomes and provide further evidence in support of the efficacy of rural collective property rights reform policies.

### Endogeneity test using Propensity Score Matching (PSM)

The discussion on endogeneity needs to be more convincing. Self-selection issues may indeed be present; however, it is crucial to clarify how these issues relate to endogeneity. Self-selection can lead to omitted variables bias, which occurs when unobserved factors influencing both the treatment assignment and the outcome are not accounted for. In this study, this bias could arise if certain unobserved characteristics of villages or farmers influence both their likelihood of participating in the reform and their economic outcomes. Specifically, the bias related to the identification strategy could stem from unobserved factors such as the entrepreneurial spirit of farmers, local governance quality, or pre-existing economic conditions. These factors might simultaneously affect the decision to engage in land reforms and the subsequent economic benefits, thus confounding the estimated effects of the reform. To address these biases, the Propensity Score Matching (PSM) method is employed. PSM is appropriate for alleviating these biases because it allows for the creation of a matched sample of treated and untreated units that are similar in terms of observed characteristics. By matching on the propensity score, it is possible to control for the observed covariates that predict treatment assignment, thereby reducing the bias due to confounding variables. PSM helps to balance the distribution of observed covariates between the treated and control groups, making the comparison more robust and reliable. This method has been widely used in observational studies to address issues of endogeneity and self-selection, providing a more accurate estimate of the treatment effect. Table 5 presents the PSM regression outcomes employing three distinct matching techniques—Nearest Neighbor Matching, Caliper Matching, and Kernel Matching. Regardless of the matching method applied, a significant positive impact of rural collective property rights reform on rural income levels was demonstrated. Specifically, the Average Treatment Effect on the Treated (ATT) was 0.259 for Nearest Neighbor Matching (Model 1), 0.318 for Caliper Matching (Model 2), and 0.308 for Kernel Matching (Model 3), with these effects statistically significant at the 1% level (***). This indicates that rural collective property rights reform significantly enhanced rural income levels, and this outcome remains robust across different matching methodologies.

**Table 5:** PSM regression results.

	Matching Method	Treatment Group	Control Group	ATT	Standard Error	T Value
Rural Income Level	Nearest Neighbor Matching	9.519	9.259	0.259***	0.079	3.29
	Caliper Matching	9.546	9.228	0.318***	0.107	2.98
	Kernel Matching	9.532	9.224	0.308***	0.116	2.65

To validate the effectiveness of the propensity, score matching results in balancing the data, a balance test was conducted. Table 6 provides the outcomes of the balance test, exemplifying the Nearest Neighbor Matching method. Prior to matching, there were some differences in the means of numerous controlled variables between the treatment group and the control group. Following matching, these discrepancies in controlled variables significantly reduced, with all biases rates falling under 10%. Additionally, the t-values from the post-matching T-test did not reject the null hypothesis of no systematic differences between the treatment group and control group, signaling a balance in controlled variables following the match. This confirmed the validity of the propensity score matching results. Therefore, through the endogeneity test using the propensity score matching method, the study suggests that rural collective property rights reform has a significant positive effect on enhancing rural income levels, and this effect remains robust across different matching methodologies. The passing of the balance test further substantiates the appropriateness of the matching methods, reinforcing the credibility of the research conclusions. These findings provide compelling evidence supporting the efficacy of the rural collective property rights reform policy.

**Table 6:** Balance test results of Propensity Score Matching (PSM) using the nearest neighbor matching method.

Control Variable	Mean before Matching		Mean after Matching		Deviation Rate (%)		T-test after Matching	
	Experimental Group	Control Group	Experimental Group	Control Group	Before	After	T	P > t
X1	51.647	50.557	51.693	51.631	17.7	1.0	0.009	0.927
X2	52.814	52.247	52.146	52.21	1.7	-0.2	-0.02	0.986
X3	483.28	568.09	481.04	487.56	-16.3	-1.3	-0.12	0.903
X4	1268.1	1370.2	1253.5	1293.8	-10.0	-4.0	-0.36	0.716
X5	14.337	7.985	10.871	10.161	15.8	1.8	0.23	0.815
X6	5.008	1.747	3.177	2.404	25.9	6.1	0.98	0.330
X7	2.840	2.945	2.856	2.898	-13.8	-5.6	-0.50	0.619
X8	0.117	0.179	0.119	0.103	-27.8	7.2	0.77	0.444
X9	0.205	0.237	0.189	0.176	-7.7	3.4	0.33	0.745

### Mechanism examination

To further understand the underlying mechanisms through which the rural collective property rights reform affects economic outcomes, it is essential to explore the mediating effects. While the baseline regression model provides an initial understanding of the direct impact of the reform, it does not capture the indirect pathways through which the reform influences the dependent variables. The mediating effect model is needed to identify and quantify these indirect effects, thereby offering a more comprehensive understanding of the reform’s impact. Specifically, the reform may influence economic outcomes through two key mediating variables: rural collective economic assets and land transfer. These two mechanisms are interconnected and can mutually reinforce each other. For instance, the enhancement of rural collective economic assets can lead to better infrastructure and services, which in turn can facilitate more efficient land transfers. Conversely, increased land transfers can generate additional income for rural collectives, further boosting their economic assets. By incorporating these mediators into the analysis, it is possible to examine how the reform’s direct effects are transmitted through these channels and how they interact with each other. This approach allows for the disentanglement of the complex relationships between the reform and economic outcomes, providing insights into the specific pathways and mechanisms at play. Tables 7 and 8 present the findings of the mechanism examination into how rural collective property rights reforms have influenced rural income levels. The research initially contemplated the total assets of village collectives as a potential mediating variable. Results shown in Table 7 indicate a significant positive effect of rural collective property rights reform (Model 3) on rural income levels (0.138***). In parallel, the total assets of the village collective (Model 2) also have a significant positive effect on rural income levels (191.434**). Furthermore, the coefficient for the village collective’s total assets (0.0002***) suggests that it plays a partial mediating role between property rights reform and rural income levels. This implies that rural collective property rights reform could indirectly enhance rural income levels by increasing the total assets of the village collective. Additionally, the study considered the role of land transfer as a mediating variable. Table 8 displays a similar pattern: rural collective property rights reform has a marked positive impact on rural income levels (0.138***), and land transfer (Model 2) also positively influences rural income levels significantly (339.355*). The coefficient for land transfer (0.0001*) also indicates its partial mediating effect between property rights reform and rural income levels. This suggests that land transfer is another important conduit through which rural collective property rights reform affects rural income levels. In both mechanism examinations, other relevant variables have been controlled to ensure accuracy. The R^2^ values of the models demonstrate the degree of fit to the data, with an R^2^ value of 0.2653 in Table 7, and 0.2653 (Model 1 and 3) and 0.1599 (Model 2) in Table 8. This signifies that while the model can account for part of the variance in rural income levels, other factors may also influence these levels.

**Table 7:** Mechanism test results for total assets of village collective.

Variable Name	(1)	(2)	(3)
	Rural Income Level	Village Collective Total Assets	Rural Income Level
Reform	0.138***	191.434**	0.138***
	(0.053)	(93.606)	(0.053)
Village Collective Total Assets			0.0002***
			(0.0001)
Control Variables Controlled	Yes	Yes	Yes
R^2^	0.2653	0.1050	0.2653

**Table 8:** Mechanism test results for land transfer.

Variable Name	(1)	(2)	(3)
	Rural Income Level	Land Transfer	Rural Income Level
Reform	0.138***	339.355*	0.249***
	(0.053)	(187.886)	(0.091)
Land Transfer			0.0001*
			(0.0001)
Control Variables Controlled	Yes	Yes	Yes
R^2^	0.2653	0.0798	0.1599

Overall, the results of the mechanism examinations reveal two potential pathways through which rural collective property rights reform can enhance rural income levels: firstly, by increasing the total assets of village collectives, and secondly, by facilitating land transfer. Both mechanisms provide indirect evidence of how rural collective property rights reform can influence rural income levels, hence validating Hypothesis 3.

### Heterogeneity analysis

Table 9 exhibits the heterogeneity results for the impact of rural collective property rights reform on rural income levels. One facet of this heterogeneity is the distinction between impoverished and non-impoverished villages. The study identified that the upliftment in rural income levels post-reform was more pronounced in non-impoverished villages. Specifically, the coefficient for the reform in non-impoverished villages stood at 0.166*** (Model 2), while that for impoverished villages was 0.104 (Model 1). This suggests a greater facilitation of income level enhancement as a result of property rights reform in non-impoverished villages, potentially due to their advantageous resources and economic conditions, which allow for a more effective ignition of rural economic vitality. Secondly, heterogeneity based on the educational attainment of village leaders was considered. The higher educational levels of village leaders proved to influence the efficacy of the reform. Villages with better-educated leaders (Model 4) yielded a coefficient for collective property rights reform at 0.193***, compared to 0.182 for villages with less educated leaders (Model 3). This indicates that leaders with higher educational qualifications may be more capable of efficiently driving reform, harnessing their knowledge and skills to generate greater economic returns for the rural community. Thirdly, regional disparities were also a significant factor. The impact of the reform varied substantially across regions, with the most significant uplift in rural income levels manifesting in the western region, where the coefficient reached 0.286*** (Model 6), compared to 0.156 for the central region (Model 5) and 0.099 for the eastern region (Model 7). This variation could relate to the lower economic development level in the western region, where the marginal effects of the reform are greater.

**Table 9:** Heterogeneity analysis results.

Variable Name	Poverty Village Heterogeneity		Education Level of Village Head Heterogeneity		Regional Heterogeneity		
	Yes	No	Low Education Level	High Education Level	West	Central	East
Rural Collective Property Rights Reform	0.104	0.166***	0.182	0.193***	0.286***	0.156	0.099
	(0.089)	(0.067)	(0.138)	(0.060)	(0.102)	(0.114)	(0.138)
Control Variables Controlled	Yes	Yes	Yes	Yes	Yes	Yes	Yes
R^2^	0.1142	0.2159	0.2588	0.2315	0.3306	0.4386	0.2984

In all models, control has been exercised for additional relevant variables to assure the preciseness of the results. The R^2^ values reflect the fit of the model to the data, with an R^2^ of 0.1142 for the impoverished village model, 0.2159 for the non-impoverished village model, 0.2588 for the less educated leader model, 0.2315 for the more educated leader model, 0.3306 for the western region, 0.4386 for the central region, and 0.2984 for the eastern region. These figures highlight variability in explanatory aptitude across different sub-samples, with the central region displaying the highest model fit. The outcomes of this heterogeneity examination reveal the diversified impacts of rural collective property rights reform on enhancing rural income levels. The effects are notably substantial in non-impoverished villages, among villages with higher-educated leaders, and in the western region. Such findings equip policymakers with the insight to pinpoint priority areas for reform implementation and adopt varied strategies under different conditions to maximize the socioeconomic benefits of reform.

## Conclusions and policy recommendations

An economic effect analysis of rural collective property rights reform reveals its positive role in promoting income increase and poverty reduction. (1) Through robustness tests, this paper has excluded the biases stemming from sample selection and the interference of outliers, affirming the reliability of the positive effects of property rights reform. (2) Correction of endogeneity issues further ensures the causal interpretability of the research findings, while mechanism tests have unveiled that property rights reforms function through two key channels: the increase of collective assets and the facilitation of land transfers. (3) Heterogeneity analysis highlights the variability in the effects of property rights reform, pointing to the potential advantages under specific conditions. (4) The successful implementation of property rights reform necessitates considering the specific context and socioeconomic conditions of rural areas. Non-impoverished villages generally possess better resources and economic conditions, allowing for a more efficacious utilization of the opportunities presented by property rights reform. Village leaders with higher levels of education may have a deeper understanding of reform policies and are likely more capable of effectively advancing the reform process. In the western region, due to the relatively lower level of economic development, the marginal effects of property rights reform could be greater, thus rendering the impetus of reform more significant.

In accordance with the aforementioned deductions, several tactical recommendations are thus presented:

Intensifying property rights reform to invigorate rural enrichment. This necessitates the expansion of rural collective property rights reform, enhancing its scope and influence. For regions where the reform has exhibited benefits, lessons learned should be collated to form reproducible, scalable models that can serve as guidance for other regions. Simultaneously, localized innovations should be supported based on the actual situations, therefore exploring reform trajectories that correspond to unique characteristics. For specific rural conditions, such as non-impoverished villages and the western region, more ambitious policies should be enacted to fully tap the marginal effects of property rights reform, thereby unlocking the intrinsic potential of rural development.

Strengthening capacity building to augment reform execution proficiency. Building the capacity of village leaders and rural management is pivotal in certifying the smooth progression of property rights reform. It is recommended to boost the breadth and depth of predefined training for these roles, particularly in legal, financial, and managerial aspects, which can consequently fortify the comprehension and execution faculties towards property rights reform.

Streamlining land rotation, enhancing the management level of collective assets: Land rotation and collective asset management are salient constituents of property rights reform, serving as pivotal routes to bolster rural revenue.

Precision policymaking through differentiated strategies: Given the heterogeneity of property rights reform outcomes, the implementation of a unified strategy is a crucial pathway to achieving precision policymaking.

Direct references to the differences in poverty status, educational level, and geographical location of rural areas should be explicitly integrated into the crafting of such reform strategies. Specific abstracts to reform policy can be further delineated in the case of impoverished villages, educational advanced communities, and regions with lower economic development indices. For example, destitute villages could benefit from more supportive provisions, while more autonomous rights can be bestowed upon communities with higher education rates. As for regions with low levels of economic development, policy bias should be significantly intensified to harness the marginal effects of property rights reform.

The execution of these strategic measures is pivotal for propelling rural collective property rights reform in a more profound and effective manner, thus establishing a robust foundation for the continuous and healthy growth of the rural economy. Additionally, such initiatives are instrumental in nurturing equity and justice within rural communities, amplifying the inhabitants’ sense of fulfillment and happiness, and contributing to the harmony and stability of rural society. It is only through the adoption of integrated and systematic reform strategies that the comprehensive and enduring success of rural collective property rights reform can be guaranteed, serving as a strong pillar in achieving the modernization of agriculture and rural areas alongside the strategic objectives geared towards rural rejuvenation.

## Supporting information

S1 File (PDF)
