# Supplementary material for: The impact of rural collective property rights reform on income and poverty reduction: Evidence from China’s rural regions
Source: PLoS One. 2024 Sep 6;19(9):e0308393. doi: 10.1371/journal.pone.0308393 (PMC11379265; doi:10.1371/journal.pone.0308393)
Supplement: S1 File — (PDF) [file pone.0308393.s001.pdf]

村代码：__________

中国社科院乡村振兴数据库建设

村调查表

省（自治区、直辖市）___________________________

市（州）： ___________________________

县（市、区）： ___________________________

乡镇： ___________________________

行政村： ___________________________

受访者姓名： ___________________________

受访者职务： ___________________________

手机号码： ___________________________

微信号： ___________________________

调查员： ___________________________

1. 基本情况

序号	问题	选项/代码	答案
1-1	地势	1=平原；2=丘陵；3=山区（注：海拔200米以下平原，200-500米丘陵，500米以上山区；也可以目测观察）4=半山区	
1-2	2019年人均可支配收入	元（注：按上报数填写；如无，按估计数填写，并备注一下）	
1-3	是否为城市郊区	1=是；2=否	
1-4	村委会距离乡镇政府距离	公里（注：按车行距离，可以用手机导航）	
1-5	村委会距离县政府距离	公里	
1-6	村党支部最近一次换届时间	YYYY：例如，2015	
1-7	村党支部书记性别	1=男；2=女	
1-8	村党支部书记年龄	岁	
1-9	村党支部书记学历	1=未上过学；2=小学；3=初中；4=高中；5=职高/技校；6=中专；7=大专；8=本科；9=研究生；10=其他（请说明）	
1-10	村党支部书记是否兼任村委会主任	1=是；2=否	
1-11	如果是，则哪年兼任？	YYYY：例如,2015（注：按首次兼任时间算起）	
1-12	担任村党支部书记已经几年	年	
1-13	有几个村民小组	个	
1-14	是否贫困村？如果否，转到下表	1=是；2=否（注：标准为国定建档立卡贫困村；之前是贫困村，后来退出的也算）	
1-15	如果是，则哪年确定的？	年	
1-16	现在是否退出贫困村	1=是；2=否	
1-17	如果退出，则哪年退出的？	年	

2.农户、人口与劳动力构成

序号	问题	选项/代码	答案
2-1	总户籍数（户数）	户	
2-2	其中：1.建档立卡贫困户数	户	
2-3	已有多少建档户出列	户	
2-4	2. 五保户户数	户	
2-5	3.低保户户数	户	
2-6	4.常年不在村的农户数	户	
2-7	村里种养专业户	户	
2-8	本村个体工商户	户	
2-9	本村电商户	户	
2-10	外来个体工（电）商户	户	
2-11	外来农业规模经营专业户（含家庭农场）	户	
2-12	户籍总人口数	人	
2-13	常住人口数	人	
2-14	其中:1.外来人口数	人	
2-15	2.60岁以上人口数量	人	
2-16	3.女性人口	人	
2-17	全村农村劳动力数（含本村与外来劳动力）	人	
2-18	其中：本村户籍（包括户籍迁出的原住民）	人	
2-19	外来常住	人	
2-20	其中：主要本地第一产业人数	人	
2-21	主要本地二三产	人	
2-22	主要外出务工（注：外出三个月以上）	人	
2-23	常年外出务工的劳动力数	人	
2-24	因疫情，2020年外出务工人数是否有变化？	1=没有；2=有所减少；3=减少较多；4=有增加；5=增加较多	

3. 土地情况

序号	问题	选项/代码	答案	相关说明
3-1	村土地总面积	亩		注：村庄总面积是指村庄界限内的生产生活地理空间，包括耕地、园地、林地、山场、水域、道路、建设用地及未利用地等内容。
3-2	其中，耕地总面积	亩		注：包括水田、水浇地和旱地。
3-3	耕地中承包到户的总面积	亩		
3-4	耕地中村集体预留的机动地总面积	亩		
3-5	其中，园地总面积	亩		注：包括果园、茶园、橡胶园以及其它园地。
3-6	其中，林地总面积	亩		注：包括乔木林地、竹林地、红树林地、森林沼泽、灌木林地、灌丛沼泽和其他林地。
3-7	其中，草地总面积	亩		注：包括天然牧草地、沼泽草地、人工牧草地及其他草地。
3-8	其中，水库、坑塘等水域总面积	亩		
3-9	其中，山林、山场总面积	亩		
3-10	其中，设施农用地总面积	亩		注：设施农用地是指直接用于经营性畜禽养殖生产设施及附属设施用地；直接用于作物栽培或水产养殖等农产品 生产的设施及附属设施用地；直接用于设施农业项目辅助生产的设施用地；晾晒场、粮食果品烘干设施、粮食和农资临时存放场所、大型农机具临时存放场所等规模化粮食生产所必需的配套设施用地。
3-11	其中，集体经营性建设用地总面积	亩		注：农村集体经营性建设用地，是具有生产经营性质的农村建设用地，包括"农村集体经济组织使用乡(镇)土地利用总体规划确定的建设用地兴办企业或者与其他单位、个人以土地使用权入股、联营等形式共同举办企业、商业所使用的农村集体建设用地，如过去的乡镇企业和招商引资用地。
3-12	其中，公益性公共设施建设用地总面积	亩		注：公益性公共设施建设用地指承载集体福利或者公共利益的各类用地，包括小学校舍、村庄福利院等
3-13	其中，宅基地总面积	亩		注：此处宅基地总面积为村民实际使用的面积，包含超标和多占部分。
3-14	其中，其他未利用地总面积	亩		注：未利用地包括荒地、荒山、盐碱地、沙地等。
3-15	截至2019年底，本村撂荒土地总面积	亩		

3.土地情况（续表）

序号	问题	选项/代码	答案
3-16	本村实有宅基地宗数	宗	
3-17	法定宅基地面积按照什么标准？	代码1	
**代码1**：1=以户定标，则跳至3-18；2=以人定标，则跳至3-19；3=其他，请说明_____			
3-18	如果以户为标准，则标准是？	平方米/户	
3-19	如果以人为标准，则标准是？	平方米/人	
**注**：法定宅基地面积标准是指政府制定的农村宅基地管理办法、细则等政策文件所规定的宅基地面积，一般为省级政府制定。			
3-19	闲置宅基地宗数	宗	
**注**：农民工全家外出打工，逢年过节短期、临时性居住的情况可不统计为“闲置”。			
3-20	闲置宅基地面积	亩	
3-21	闲置宅基地中，因本村农户向他人出租房屋而发生的宅基地流转宗数	宗	
**注**：①“他人”，此处既包括本村农户、也包括本村外的农户或其他人群，因此可能存在违法出租的情形，但也需要统计在内；②出租，是指农户有限期限内流转宅基地使用权，而非永久性转让该宗宅基地使用权。			
3-22	闲置宅基地中，因本村农户向他人出租房屋而发生的宅基地流转面积	亩	
3-23	闲置宅基地中，本村农户有偿转让宅基地宗数	宗	
**注**：此处有偿转让，是指该农户在转让该宗宅基地后永久性丧失对该宗宅基地的使用权。			
3-24	闲置宅基地中，本村农户有偿转让宅基地面积	亩	
3-25	近三年（2017-2019）本村农户自愿退出宅基地宗数	宗	
**注**：包含自愿有偿退出和自愿无偿退出。			
3-26	近三年（2017-2019）本村农户自愿退出宅基地面积	亩	
3-27	近三年（2017-2019）本村依法收回的宅基地宗数	宗	
3-28	近三年（2017-2019）本村依法收回的宅基地面积	亩	
3-29	截至2019年底，耕地流转总面积	亩	
3-30	流转给其他普通农户	亩	
3-31	流转给家庭农场/种粮大户	亩	
3-32	流转给合作社	亩	
3-33	流转给企业	亩	
3-34	通过“反租倒包”方式流转的土地面积	亩	
注:“反租倒包”方式，是指村集体统一将农户承包地收回整理，并出租给本村农户或其他土地经营者的方式。			
3-35	2019年，本村耕地流转平均价格	元/亩年	
	耕地种植结构		
3-36	粮食作物生产	亩	
3-37	经济作物播种面积	亩	
3-38	设施农业生产	亩	
3-39	林果茶露天生产	亩	
3-40	其他生产1（___________）	亩	
3-41	其他生产2（___________）	亩	
3-42	其他生产3（___________）	亩	
3-43	农闲平均雇工工资	元/日	
3-44	农忙平均雇工工资	元/日	

4. 集体经济发展

序号	问题	选项/代码	答案
4-1	是否完成农村集体经营性资产产权制度改革	1=还没开始；2=正在进行； 3=已经完成	
4-2	如果完成，是否设置集体股股权？	1=是；2=否跳至4-6	
4-3	如果有，则集体股的股份占总股份的比例是多少？	%	
4-4	是否进行了集体股股金分红？	1=是；2=否	
4-5	如果是，集体股与个人股的分红率是否一样？	1=是；2=否	
4-6	村集体资产总额	万元	
4-7	生产性固定资产原值	万元	
4-8	存款余额	万元	
4-9	对外投资余额	万元	
4-10	现金余额	万元	
4-11	经营性资产净值	万元	
4-12	借出款余额	万元	
4-13	待收款余额	万元	
4-14	其他1（__________）	万元	
4-15	其他2（__________）	万元	
4-16	其他加总	万元	
4-17	村集体负债总额	万元	
4-18	企业上缴款	万元	
4-19	投资收益	万元	
4-20	补助收入	万元	
4-21	其他1（__________）	万元	
4-22	其他2（__________）	万元	
4-23	其他加总	万元	

4. 集体经济发展（续表）

4-24	村集体年内支出合计	万元	
4-25	经营性总支出	万元	
4-26	其中：为农户提供生产服务支出	万元	
4-27	购置生产性固定资产支出	万元	
4-28	农田水利基本建设支出	万元	
4-29	公益事业支出	万元	
4-30	其中：农户节日福利支出	万元	
4-31	五保户烈军属老人等补助支出	万元	
4-32	文化支出	万元	
4-33	医疗卫生健康费用支出	万元	
4-34	垃圾清运等环保费用支出	万元	
4-35	社会治安费用支出	万元	
4-36	村庄道路建设与维护支出	万元	
4-37	行政管理费支出	万元	
4-38	其中：招待费支出	万元	
4-39	其中：干部工资和补贴支出	万元	
4-40	其他支出	万元	

5. 农业生产经营

序号	问题	选项/代码	答案
5-1	合作社数量（备注：按合作社实际数量算，非本村发起人也算）	个	
5-2	合作社类型（可多选）	1=农产品销售；2=生产服务类（农资、农机、植保、土地流转等）；3=农产品加工；4=劳务服务	
5-3	参加农户数	户	
5-4	合作社及其社员经营面积	亩	
5-5	带动农户数量	户	
5-6	本村是否有乡村旅游	1=是；2=否	
5-7	主要旅游项目	1=体验农业；2=自然风光；3=特色小吃；4=特色文化；5=特色产业；6=其他（请说明）________	
5-8	当年接待游客数量	人次	
5-9	其中：本村留宿数量	人次	
5-10	本村参与乡村旅游产业户数	户	
5-11	本村最开始乡村旅游项目的年份	YYYY：例如，2010	
5-12	本村是否有农户经营网店	1=是；2=否	
5-13	经营网店的农户数量	户	
5-14	销售的主要农产品种类	文字说明	
5-15	电商销售量最高的农产品品类，占总产量的比例	%	
5-16	本村是否有电商服务站或产品代售点	1=是；2=否	

6. 农村事业(注：下表中的多选，请按照重要程度排序)

序号	问题	选项/代码	答案
6-1	本村集中供水覆盖的农户比例	%	
6-2	饮用“安全卫生水”的户数 （无杂质、无色无味且长期饮用无不良反应）	户	
6-3	饮用自来水的农户比例	%	
6-4	全村已用电的农户比例	%	
6-5	村与组之间的道路是否是硬化道路	1=是；2=否	
6-6	全村已连宽带户数	户	
6-7	是否可以实现有快递到户（包括另付费情况下）	1=所有户都能到；2=仅到部分户；3=不能到户	
6-8	若到部分户/不能到户，则本村否有快递提货点	1=是；2=否	
6-9	是否通过微信等建立全村性信息发布和交流群	1=是，全村群（包括村民小组群）；2=没有交流群	
6-10	近三年是否遭受自然灾害	1=是；2=否	
6-11	本村是否设置垃圾桶	1=是；2=否	
6-12	垃圾桶数量（个数）	个	
6-13	本村是否有专人负责垃圾清运	1=是；2=否	
6-14	本村保洁员数量	人	
6-15	本村是否有废旧农膜或农药包装物回收点	1=是；2=否	
6-16	您村近5年是否有过工业污染（工业垃圾或者工业污水）？	1=是；2=否	
6-17	本村是否有生活污水管网设施	1=是；2=否	
6-18	2019年内全村红白喜事数量	数	
6-19	每起红白喜事酒席规模	桌数	
6-20	平均随礼金额	元	
6-21	年内发生各类刑事犯罪案件次数	次	
6-22	信仰“耶稣”（或天主教、东正教）的人数	人	
6-23	信仰伊斯兰教的人数	人	
6-24	信仰佛教的人数	人	
6-25	信仰其他宗教人数	人	

代码1：1=起居照料；2=上门看病送药；3=精神慰藉聊天解闷；4=日常购物；5=组织社会和娱乐活动；6=提供法律援助（维权）；7=提供保健知识；8=处理家庭邻里纠纷；9=其他（请说明）__________

7. 养殖情况

序号	问题		单位	答案
7-1	肉牛	大规模(大于500)	户	
7-2		中规模(50-500]	户	
7-3		小规模(10-50]	户	
7-4		散养（小于等于10]	户	
7-5	奶牛	大规模(大于500)	户	
7-6		中规模(50-500]	户	
7-7		小规模(10-50]	户	
7-8		散养（小于等于10)	户	
7-9	肉羊	大规模(大于500)	户	
7-10		中规模(100-500]	户	
7-11		小规模(50-100]	户	
7-12		散养（小于等于50)	户	
7-13	生猪	大规模(大于1000)	户	
7-14		中规模(100-1000]	户	
7-15		小规模(30-100]	户	
7-16		散养（小于等于30)	户	

8. 村级党组织建设

序号	问题	选项/代码	答案
8-1	本村党组织的名称是	1=党支部；2=总支部；3=党委或分党委	
8-2	若回答2或3，请问本村的基层党组织数量	个	
8-3	全村党员人数	人	
8-4	其中：男性党员	人	
	本村党员的年龄构成		
8-5	其中：40岁及以下	人	
8-6	41-59岁	人	
8-7	60岁及以上	人	
8-8	本村党组织（党支部）人员的平均年龄	岁	
8-9	2017年以来新发展的党员人数	人	
8-10	2017年以来新发展党员的平均年龄	岁	
8-11	2017年以来递交入党申请书人数	人	
8-12	最近一次全村党员大会召开时间	格式：如2019.09	
8-13	每年召开几次村级党组织支委会会议	次	
8-14	村级党组织（党支部）最近一次换届时间	格式：如2019	
8-15	任书记前的个人身份（可多选）	代码1	
8-16	村党组织（党支部）书记是否有外出打工经历	1=是；2=否	
8-16	去年村级党组织（党支部）书记的收入（含工资、补贴和上级奖励）	万元	
8-18	其中：由财政支付的工资	万元	
8-19	2019年村级党组织（党支部)的经费	万元	
8-19	其中：来自上级财政拨款	万元	
**代码1**：1=生产经营大户；2=个体工商户；3=企业主；4=退伍军人；5=村两委干部；6=村医/农技等专业人员；7=政府干部；8=其他（请说明）__________			
